# Anonymised human location data in England for urban mobility research

**DOI:** 10.1038/s41597-025-06323-8

**Published:** 2025-11-26

**Authors:** Chen Zhong, Zhengzi Zhou, Nilufer Sari Aslam, Yikang Wang, Adham Enaya

**Affiliations:** Centre for Advanced Spatial Analysis, University College London, London, W1T 4TJ UK

**Keywords:** Geography, Research data

## Abstract

The rapid expansion of sensor technologies and location-enabled mobile applications has greatly advanced the study of human mobility. Recently emerged sources like mobile app data offer outputs similar to traditional datasets—trip chains, flows, and indicators—but the methods and decisions used to process such data often vary across location, time, and policy context and remain poorly documented and insufficiently transparent. This variability necessitates tailored data processing and validation approaches, which remain underexplored in existing literature. This study aims to provide a reproducible and replicable framework for processing location-points data, using a case study of anonymised mobility records collected in November 2021 across England. We describe a modular workflow and multi-stage validation techniques that enhance the reproducibility of stay-point detection and activity labelling. Furthermore, we demonstrate how the proposed framework can generate reliable mobility indicators and origin-destination flow matrices for broader research applications. The resulting datasets—including sampled anonymised trajectories and full origin-destination flow matrices—are publicly available for research purposes, with updates to code and methodology hosted on GitHub and Zenodo.

## Background & Summary

As demand for mobile services has multiplied over the last decade^1^, the market is expected to continue growing rapidly, with people seeking high-quality mobile services wherever they live, work, and travel. Simultaneously, mobile services generate large volumes of fine-grained data through various sensors. Among these, mobile app data—collected via software applications (‘apps’) installed on smartphones and other wearable devices^2–4^ —has emerged as a promising source. Mobile app data is acquired with users’ opt-in consent for location tracking. Nonetheless, passive data collection still raises legitimate ethical concerns, such as transparency, purpose limitation, and data retention practices, which must be considered carefully. Mobile app data offers several advantages but is also marked by biases rooted in smartphone adoption rates, market penetration of individual apps, users’ preferences (e.g., app selection, usage intensity, willingness to share location), and geographic differences in data quality. These issues affect the reliability and representativeness of the data, yet the extent of these limitations and best practices for mitigating them remain underexplored.

The value of human location data has been demonstrated in numerous urban applications, as it is essential for addressing individual needs, especially in the era of people-centric urban planning. Despite its short history, mobile in-app data has also been applied in a number of recent studies to investigate mobility and activity patterns^5,6^, socio-spatial inequalities^7^, disaster management^8^, urban and regional development^9^, economic activities^10^, informing public health policy^11^ and the generic laws and mechanisms of cities^12^. In parallel, data has become a strategic asset in the digital economy, characterised by growing markets. However, mainstream data providers (e.g., SafeGraph, CARTO, Cuebiq) still focus on conventional products such as POIs, while emerging location-based datasets remain less visible, often due to regulatory challenges (e.g., GDPR), limited transparency, and nascent business models.

Given the overall landscape of mobile in-app data research and commercial innovation, it is time to explore its full potential, with a clear awareness of its limits. Meanwhile, the open science movement has significantly altered the research culture, facilitating an open and collaborative scientific community for information sharing and collective efforts, particularly during the pandemic. Open data sets are generated from mobile app data. For instance, daily time-series of three different aggregated mobility metrics in Italy were shared for monitoring the impact of the lockdown^13^; multiscale origin-to-destination (O-D) population flows across the US has been published for monitoring epidemic spreading^14^; a city-scale and longitudinal dataset of anonymised human mobility trajectories in Japan has been shared for benchmarking human mobility predicting models^4^. Over 11 billion geolocated cell phone records from Greater Mexico City were analysed for dynamics before, during, and after COVID-19^15^. In the UK, time-series counts data are made open at an aggregated level through national research facilities, e.g., the Consumer Data Research Centre (https://www.cdrc.ac.uk/about/). While this open data has shed light on equal access and collective intelligence, there are still obstacles to maximising the usage and impact of the data. In particular, a unified technical framework for data processing is missing, which makes it difficult for data exchange and integration across borders.

To promote the research community around mobile data for urban mobility studies, we introduce an openly available dataset at a finer level that provides anonymised trajectory data for the Greater London Area and national origin-destination (O-D) data at a fine geographical scale (i.e., MSOA level census tracts), considering data bias (e.g., population coverage) and usability, as well as potential applications for urban mobility. The methods and technical validation presented in this paper contribute as a reproducible framework for processing similar types of location points data, detailing the engineering workflow and multi-stage validation techniques.

## Methods

The raw data used as input are from a UK-based data service company – Locomizer (https://locomizer.com/), who license mobile GPS data sourced from 200 mobile apps and pre-processed data (e.g., anonymisation using a cryptographic hash function, filtering noisy points and aggregation) to ensure the data adheres to local privacy regulations such as GDPR and contractual obligations with the suppliers. Although the information generated by user interactions with mobile applications could be rich, including user behaviour and device information, the data we used is simple and kept minimal attributes to minimise ethical risks and to be in line with other primary streaming automatic human mobility data (e.g., smart card data, tweets).

We used the data from November 2021 to demonstrate the workflow and share regenerated datasets for research use. We purposely selected this month as it represented a relative return to normal daily mobility following the major lifting of nationwide COVID-19 restrictions in England earlier in the year. However, we acknowledge that certain public health guidelines—particularly those protecting vulnerable populations—were still in place due to the emergence of the Omicron variant. This context should be taken into account when interpreting mobility patterns during this period. It is not a holiday season and has no bank holidays. However, the Ultra Low Emission Zone (ULEZ) expansion was implemented on 25 Oct 2021, which may bring some unknown variability to the mobility patterns. Overall, the mobile app dataset for November records a total of 793,502 active devices, covering approximately 1.028% of the national population. Coverage rates vary slightly across regions—for example, the Greater London Authority (GLA) area shows a coverage rate of 1.021%. Among all recorded devices, 628,649 (80%) have valid data for at least 14 consecutive days. In our analysis, we consider a unique device ID as representing a single user. However, we recognise that this is a simplification, as it is common for a user to possess more than one device. Readers referring to our framework should keep in mind that this limitation is a remaining issue and is not addressed in our data preprocessing. A substantial number of location points per device enable reliable activity identification and home–work detection. More statistics, including user counts, active user counts, and signal counts, are summarised in Figure S1. Additional details on data bias in terms of representativeness of population is presented in Figure S4. The original location points data has minimal attributes, including anonymised device ID, latitude, longitude, and time tag. It is worth noting that the method below was applied to the full datasets, and plots are also generated from the full datasets, not sampled data.

**Fig. 1:**
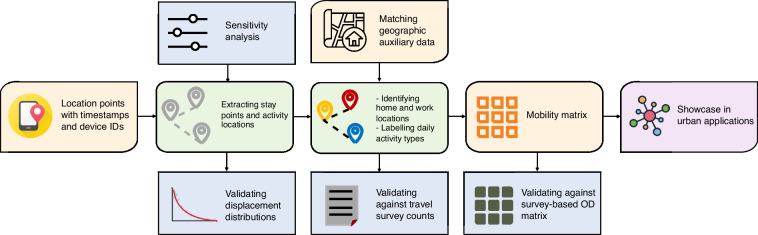
The overall data processing workflow. The orange boxes demonstrate input and output data. The green boxes demonstrate key processing steps, which are detailed in this method section. The blue boxes are add-ons that perform optimisation through sensitivity analysis and validation at each intermediate stage, documented in the technical validation section. The final part is a showcase of urban applications we considered as an extra validation at the application level, included at the end of the technical validation section.

Location data is a widely researched area with input from various domains (e.g., computer science, geography, urban planning, physics). The terminologies used may have different meanings in varied domain contexts. Therefore, we have defined terminologies in Table S1 for a unified understanding. Based on this, the overall data processing framework is presented in Fig. 1, which illustrates detailed steps from original data sets to regenerated open-access data sets. The first step is to extract stay points by filtering over-sampled noisy points. The second step combines spatial contextual information (i.e., POIs) and temporal patterns (e.g., visiting time) to infer activity types. It is possible to infer travel models by matching transition points with road networks. However, this is not scoped in this paper and is left for future updates. The final step summarises the processed data into commonly used data forms, e.g., OD flow matrix, trajectories and counts. Each intermediate result was validated with details presented in the later section of technical validation.

**Extracting stay points and activity locations** by clustering methods and sensitivity analysis: Extracting stay points removes oversampled, noisy points—redundant location records caused by high-frequency sampling—while retaining only the essential locations that define a trajectory. The most used method is spatial clustering of sequential points. We eventually adopted the Infostop Python package^16^, seeing its advantages in simplicity and computation efficiency in its fast C +  + module. Infostop is a generic stop detection framework to transform dense and rich location time series into sequences of events. In our context, events are equivalent to activities. While Infostop is effective in extracting stay points, the effectiveness is still largely affected by some self-defined critical parameters, i.e., stay as periods when an individual does not stray further than a maximum distance r_1__max_ for a minimum duration t_min_. Previous works use spatial clustering or rule-based methods to set distance thresholds from 50 to 500 m based on expert knowledge^17–21^. Specifically, we performed a four-metric sensitivity analysis (Figure S2) varying r_1_ from 0–300 m: unique-label diversity rises to a maximum near ~80 m, while very small thresholds (<25–30 m) over-fragment stays and inflate stop counts; accordingly, we set r_1 _= 50 m as a pragmatic compromise that suppresses GPS jitter, preserves near-peak spatial diversity, and avoids premature merging of distinct places. For the temporal threshold, we adopted $({t}_{min}=1\,min)$, following recent literature demonstrating that short-duration stops are increasingly relevant in high-frequency app-based mobility data^22^.

### Identify home and work locations

As implemented in most literature, we applied a simple rule-based classification to identify home and work locations. For each device ID, we take the stay point, recorded within a defined temporal window (i.e., 9 PM to 7 AM), which has the longest duration of stay compared to other location points. Similarly, a work location is identified from the non-home candidate locations for only weekdays with the longest stay (i.e., 8 AM to 17 PM) and the maximum stay duration. In our dataset, the maximum duration values are unique across individuals, so each classification results in a single unambiguous home or work location. We acknowledge the limitations of rule-based classification and suggest future work may incorporate contextual features and learning-based approaches to improve precision.

### Labelling activity types

We defined six routine activity types (i.e., home, work, education, eating and drinking, shopping type 1, shopping type 2, entertainment, and others) and labelled all detected stays to one activity each. Dividing shopping activities into two categories (i.e., frequent small consumptions and infrequent large consumptions) aligns with the literature due to behavioural differences^23^. Home and work activities are labelled for any stays at home and work locations by default. Other than home and work activities, the other activity types were labelled using joint probabilities of spatial and temporal features commonly used in the relevant literature^24,25^. We factored spatial features by counting urban context around stay points, delineated by Points of interest (POI) collected from the Ordnance Survey (https://www.ordnancesurvey.co.uk/). In particular, a buffer area (d = 500 meters) is created around each stay point. The probability of attending certain categories of POIs (in Table S2) is calculated (detailed in Supplementary Equation 1) by a Huff model^26^. Besides, temporal features were measured as the probability of activity starting time in an hourly interval over an average day from 6:00 to 24:00. For each type of activity, we draw temporal signatures inspired by the literature^23^ and customised by local travel surveys and time-use surveys (detailed in Table S3). The one with the highest joint probability is considered the labelled activity.

## Data Records

The dataset is deposited on Zenodo (10.5281/zenodo.13327082) and is also frequently updated on GitHub(https://t.ly/dzlzB). This dataset’s records are composed of two main products: anonymised trajectories from 5000 randomly sampled users in the Greater London Area (GLA) and the national OD matrix at level 9 hexagon in the h3 geospatial indexing system (https://h3geo.org/) and the MSOA levels of the UK 2021 census geography (https://geoportal.statistics.gov.uk/).

**Trajectory datasets** contain the activity-trip-chain of 5,000 individuals who travelled across the GLA in November over 30 days. For ethical considerations, each record (row) refers to an observation of a device (individual), which consists of the following columns:

- The device ID is the unique identifier of the mobile phone user
- Start time – is the timestamp of the observation sampled into 15-minute intervals.
- End time – is the timestamp of the observation sampled into 15-minute intervals
- Location - UK census tract – MSOA
- Activity label
- Duration – in minutes
  **O-D matrix data** are the national O-D matrix, which includes all trips across England, not only those into or out of the Greater London Area (GLA). This includes intra-city, inter-city, and cross-regional flows based on all observed movements during November 2021. It is provided in two files. One file contains travel-to-work trips only, and another includes all trips from all observations. Both O-D matrices are summarised at aggregated spatial units (i.e., hexagon and MSOA). The O-D matrix is in edge list format and contains three columns.
- Origin MSOA ID
- Destination MSOA ID
- Number of trips

## Technical Validation

As demonstrated in Fig. 1, we conducted intermediate validation at various stages to ensure the quality of the processed data. We consider it essential for maintaining the transparency and reproducibility of the proposed workflow, and potentially useful for identifying the cause of data bias. To brief, the below subsections report simple statistics of detected stay points which form the trajectories; validated detected home and work locations against census data; and tested the usability of O-D flows via two common applications in urban geography.

### Simple statistics of processed data

**Fig. 2:**
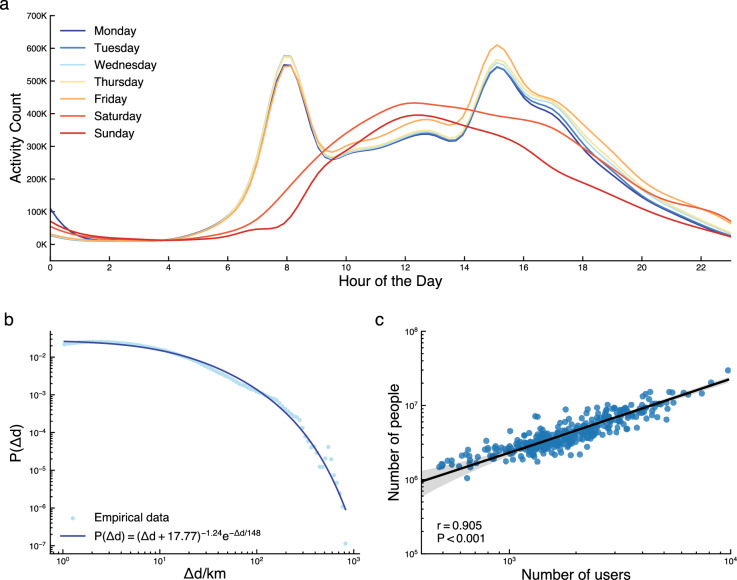
Statistical overview of processed data. (**a**) Average hourly activity counts in November 2021. (**b**) Displacement of activity locations fitting into a truncated power law. (**c**) LAD-Level Correlation between the number of residents detected from Mobile Phone App Data and reported from Census Data.

Figure 2(a) presents a simple statistical overview of processed points data (i.e., stay points without labelling activity types). The average counts of activities were summarised by starting time for each day of the week. All weekdays (Monday through Friday) show similar activity patterns with two peaks: one around 8-9 AM and another around 3-5 PM. In contrast, weekend activity patterns have one peak around midday. While Saturday presents slightly higher activity counts, the lowest activity counts are captured on Sunday throughout the day. The following three sub-sections present the validations introduced in Fig. 1.

### Distance distribution between each consecutive stays

Infostop generates stays by aggregating stationary points even though sensitivity analysis was performed to ensure that optimised parameters were set. We further validated that the generated stay points collectively follow the universal distribution. For this purpose, we measured the displacement distance between each consecutive stay, denoted as Δd. This distribution of displacement shall distinguish different types of diffusion processes, such as Lévy flights and random walk models. Although log-normal and exponential distributions have also been reported to fit well in specific datasets, the power-law distribution has proven more suitable for describing movement patterns over substantial distances. Pioneering studies utilising banknote tracking^27^ and mobile phone call records (CDRs)^28^ have demonstrated that a truncated power-law can approximate displacement distribution. In our analysis (plotted in Fig. 2(b)) the distances ranged from 1 km to 1000 km, and the fitted beta value of 1.24 aligns with the empirical range summarised in a detailed research review^12,29^. This consistency underscores the robustness of our findings in characterising human movement patterns.

### Correlation with usual residents, England: Census 2021

Figure 2(c) illustrates the Local Authority Districts (LAD)-level Pearson correlation between two datasets, namely the number of users with home locations identified from the mobile app data and the number of usual residents estimated in the UK census 2021 (https://www.nomisweb.co.uk/sources/census_2021_bulk). The Pearson correlation coefficient is 0.905, indicating a decent representation. However, when we zoom into smaller areas, the correlation decreases to 0.52. The limitation is somewhat anticipated. The detected home locations are based on simple rules, which makes it especially challenging to capture full working scenarios (e.g., night-time workers and mobile workers). Tuning the parameters used in the rule-based identification may slightly decrease/increase the numbers. Still, our sensitivity analysis shows no significant improvement. Figure S3 has further discussed the issue and bias with statistics of hourly user counts of stay location type for each day of the week. The diversity and variability of working patterns have grown significantly in recent years, particularly post-COVID. Developing a comprehensive approach to identifying irregular home and working patterns will be one of the key topics in our improvements.

### Correlation with travel to work, England: Census 2021

**Fig. 3:**
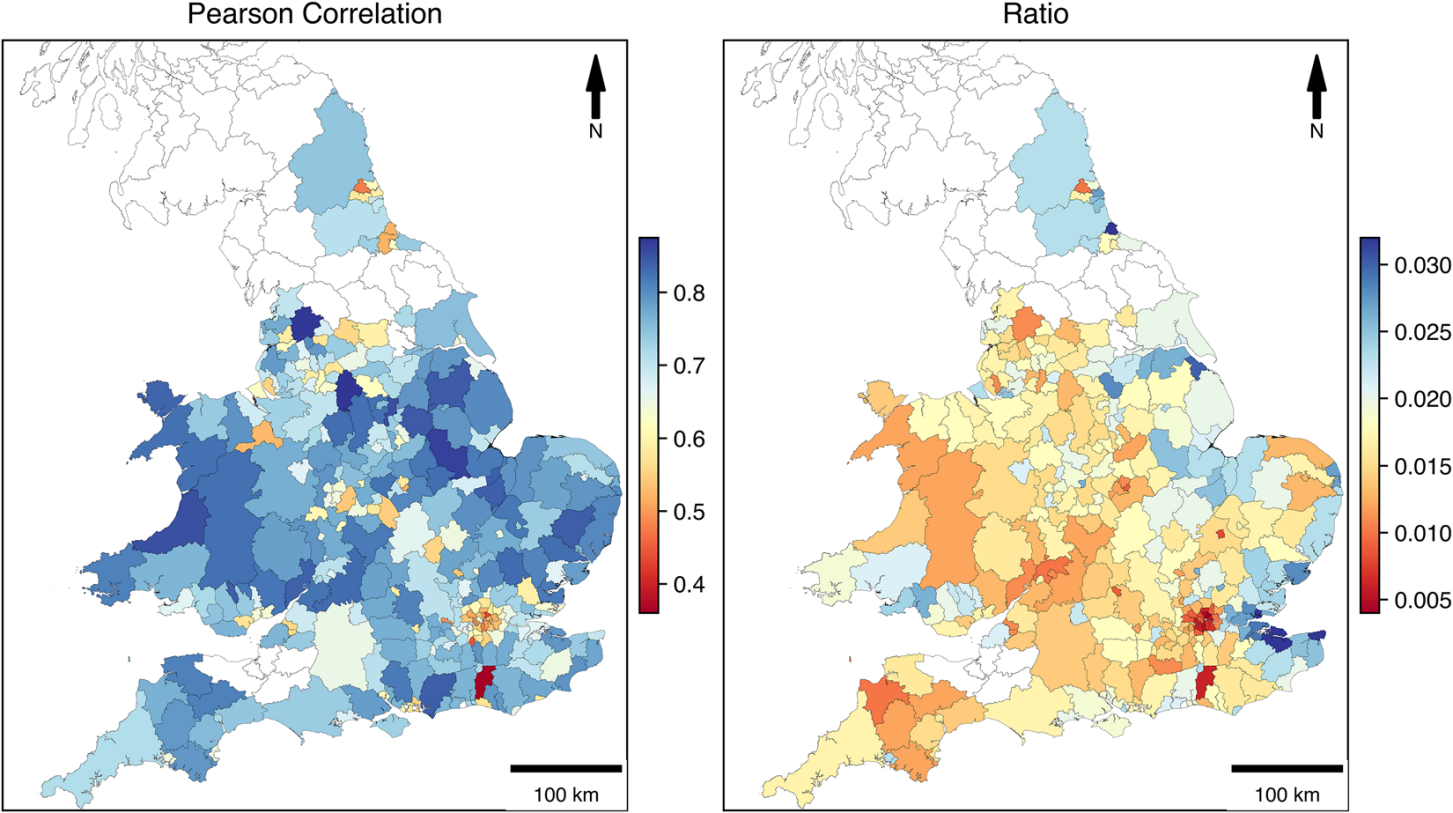
Pearson correlation and Ratio between travel-to-work trip detected from mobile app data and census data at LADs in England.

We compared our derived travel-to-work O-D matrix with multiple correlation measurements (Pearson, Spearman, and ratio as a measure of population penetration) at two levels – the Local Authority District (LAD) and the Middle layer Super Output Areas (MSOAs). The census travel-to-work data was collected through a combination of self-reported responses to specific questions related to commuting patterns and details about the time and distance. The Pearson correlation of the O-D matrix aggregated at the LAD level is 0.95, which shows good data representativeness. For MSOA level validation, we took the entire England area but grouped MSOAs by upper-level LADs to understand the variabilities across areas. In total, 331 LADs were broken down into 7264 MSOAs. To stabilise variance and handle zero values, a log1p transformation (log(1 + x)) was applied prior to correlation analysis (See Figure S5). This transformation prevents undefined values for zeros while preserving the distributional properties of the data. As reported in the Table 1, for the LDAs (311 areas out of a total of 331 LADs) with significant data records and more than one MSOA, the Pearson correlation shows decent results that range between 0.38 and 0.87 with an average of 0.7. Figure 3 provides further information about the spatial distribution. A further investigation to examine how mobility intensity relates to population is provided in Figure S6, where we applied a simple scaling index that relates the distribution of users/trips to population at the MSOA level. The spatial pattern is consistent with urban structure and data generation.

**Table 1:** Statistics of correlation between MSOAs grouped by LAD for both the census 2021 dataset and the mobile app dataset.

	Pearson	Spearman	Census (C)	Mobile (M)	Ratio (M/C)
**mean**	0.704	0.646	64944.511	1044.029	0.016
**std**	0.095	0.116	45228.264	760.316	0.005
**50%**	0.718	0.653	51323	790	0.016
**75%**	0.778	0.74	80649.5	1298.5	0.019
**max**	0.873	0.913	355566	5780	0.031

### Validation in the context of example urban applications: spatial interaction model

**Fig. 4:**
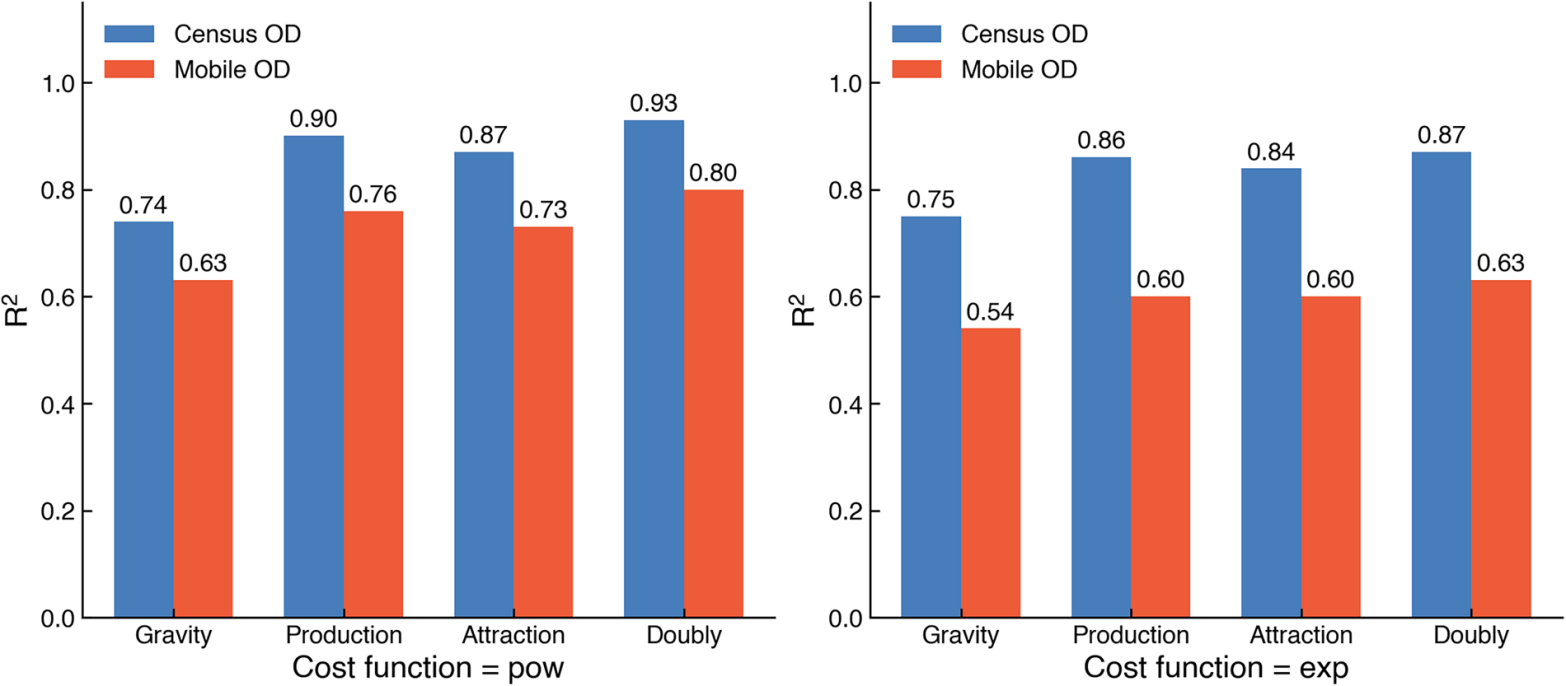
Plotting R² for different models presents the trends and consistency of goodness of fit across different models.

The processed data, while not perfectly valid as documented, proves to be highly beneficial for various urban applications, especially at aggregated scales covering large areas. Here, we compared the travel-to-work data from the mobile app and census in the context of spatial interaction application. The four variants of the spatial interaction model were employed, including (unconstrained) gravity, production-constrained, attraction-constrained, and doubly constrained models. The model parameters (*k*: balancing factor, *μ*: production, *α*: attraction, *β*: for distance decay) were estimated, with two forms of distance decay functions (i.e., power and exponential) using two sets of travel-to-work data. As shown in Fig. 4, the performance of these models, measured by the R² values, exhibits a strong correlation between the mobile app-derived flows and the census-based flows. This consistency suggests that, despite inherent biases in mobile app data, the spatial interaction models capture comparable trip distribution patterns across both datasets, reinforcing the dataset’s utility for large-scale mobility modelling.

### Validation in the context of example urban applications: spatial structure

**Fig. 5:**
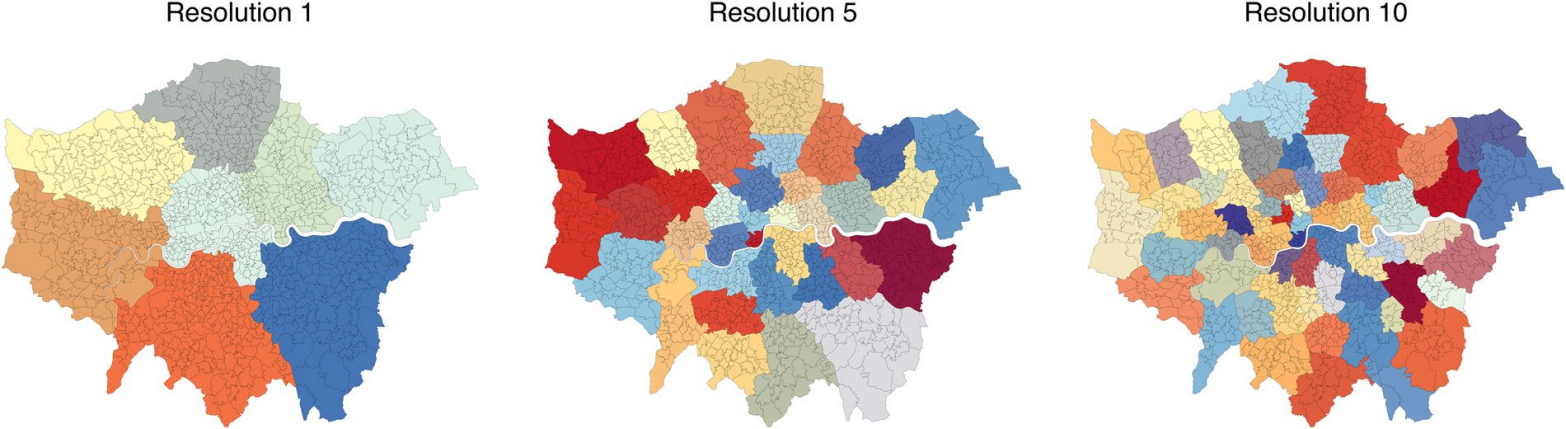
Modularity-based community detection was applied to O-D data of all trips, delineating urban functional zones at different spatial scales.

Another commonly implemented application is to detect functional spatial structures based on flow data using community detection. In network science, a community refers to a sub-network that is dense internally and sparse externally^30^; revealing these communities allows us to understand the urban structure more intuitively. A further intuitive assumption is that urban networks are organised into hierarchically distinct communities, meaning that any given scale of community can be subdivided into smaller communities, which can be further subdivided, and so on^31^. One of the most widely used and arguably most universal methods is modularity maximisation. In this study, we apply modularity-based community detection to the mobility network at three different resolutions (Fig. 5). The identified communities demonstrate clear clustering patterns of urban spatial units, which largely align with known administrative boundaries and socio-economic divisions. Importantly, this analysis serves a dual purpose: (1) it illustrates a practical application of the dataset in uncovering meaningful urban structures, and (2) it provides an internal validation of the data’s ability to capture coherent spatial patterns consistent with expected urban morphology. The coherence between detected communities and established city boundaries supports the dataset’s structural plausibility for spatial interaction research.

### Limitation and future work

Automatically collected human location data inherently contains biases rooted in how it is generated, particularly when sourced from mobile apps. Users of the shared datasets should carefully consider these limitations, in addition to complying with GDPR requirements. First, mobile app location data is recorded only when users consent, location services are enabled, and specific apps are actively used. Unlike vehicle GPS trackers with continuous coverage, mobile app data result in fragmented and irregular trajectories. These datasets should be treated as a sample of activities and trips, not as complete travel diaries. As such, derived mobility patterns reflect user behaviour selectively and may underrepresent specific groups, such as older adults, individuals with lower smartphone usage, or those residing in rural areas. Second, spatial representativeness varies across different levels of aggregation. Our validation indicates that data reliability improves at higher spatial scales (e.g., LAD level), while greater bias and variability are observed at finer resolutions (e.g., MSOA level), particularly in less populated or socioeconomically distinct areas. This should be considered when applying the data to small-area mobility analyses or when interpreting regional disparities. Third, it is worth emphasising that each step in the workflow can be improved and should be updated as data (e.g., mobile app data, census data, other supplementary data sets) of better quality, new techniques and methods emerge and are tailored for specific applications. The parameters and methods we demonstrated may serve basic needs, but are not always optimised. For instance, using hourly time windows may not be effective for travel mode detection, which requires finer temporal resolution. Fourth, to protect privacy, we have applied spatial and temporal aggregation to avoid re-identification risks. While this reduces granularity, it enables safe use for mobility research, urban planning, and comparative analysis across cities. Finally, despite these limitations, we believe the dataset contributes to promoting open science in mobility research. We will continue updating the dataset and sharing improvements via our GitHub page to enhance transparency and utility for the broader research community.

### Ethical declarations

The study has been ethically approved by UCL’s Research Ethics Committee (Ethics Application 21949/001).

## Supplementary information

Supplementary Information

## Data Availability

The dataset is deposited on Zenodo (10.5281/zenodo.13327082) and is also frequently updated on GitHub (https://t.ly/dzlzB).

## Code availability

We provide a Github repository (https://t.ly/dzlzB). Code was not generated for this work.
