# Supplementary material for: Anonymised human location data in England for urban mobility research
Source: Sci Data. 2025 Nov 26;12:2040. doi: 10.1038/s41597-025-06323-8 (PMC12749838; doi:10.1038/s41597-025-06323-8)
Supplement: Supplementary file 1 — Supplementary Information [file 41597_2025_6323_MOESM1_ESM.docx]

**Figure S1** Hourly number of device IDs (users) and points (records) in the dataset November 2021


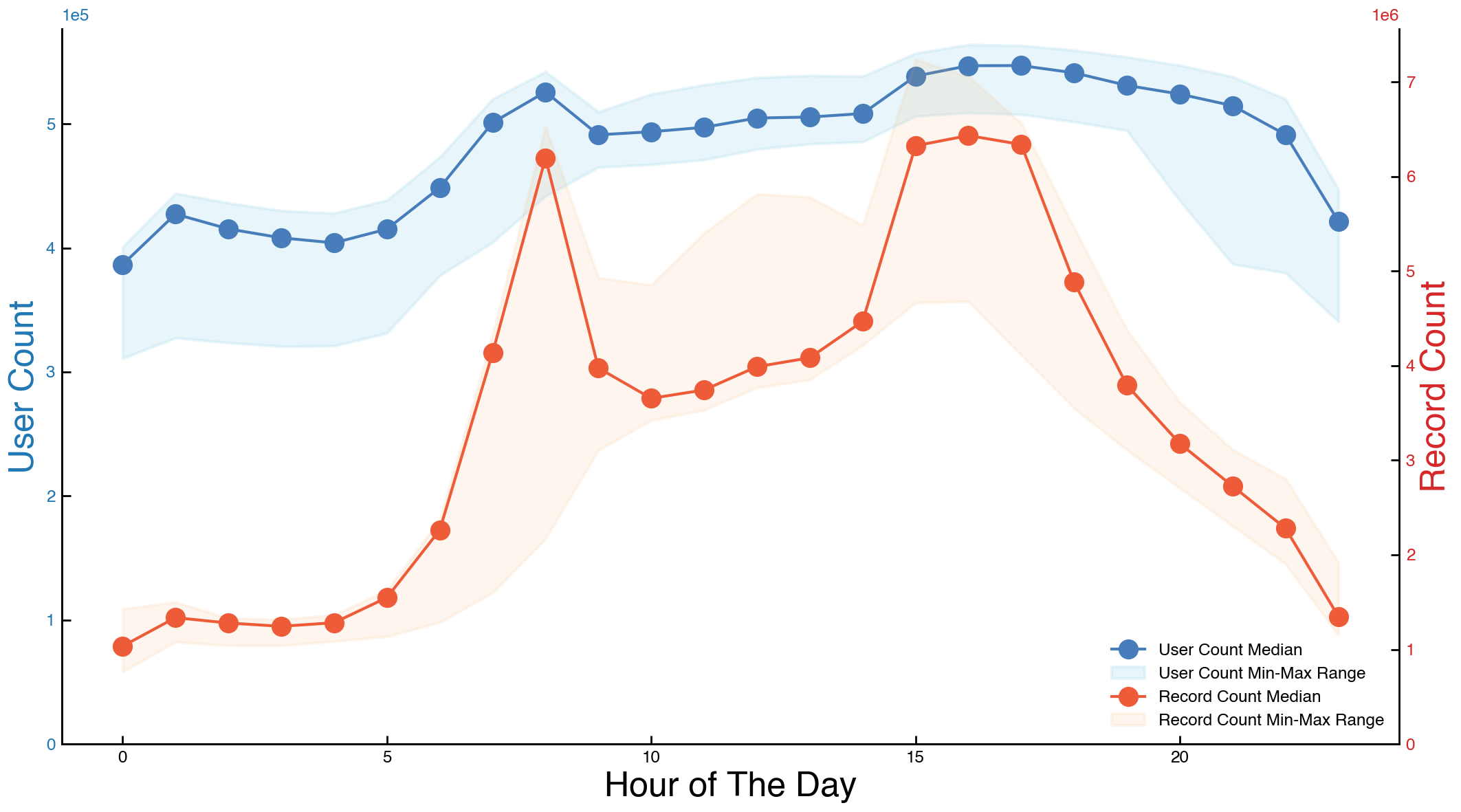


Figure A shows the hourly variation in the number of unique users (blue, left axis) and the number of location records (red, right axis) observed across all days in November 2021. The lines represent the median value per hour, while the shaded areas show the minimum–maximum range.

We observe that user availability remains relatively stable throughout the day, peaking slightly between 8 AM and 7 PM. In contrast, the number of records per hour exhibits a pronounced bimodal pattern, with peaks around commuting hours (8 AM and 5–6 PM), suggesting increased sampling activity or movement events during these times. This discrepancy highlights that user count alone does not reflect activity intensity and supports the need to consider both dimensions for mobility analysis.

**Table S1.** Defining critical terminologies

| **Location points:** | Sometimes called events, are GPS points recorded as latitude and longitude in the raw mobile app data. Stay: is extracted from a series of stationary points; in other words, a stay is defined as a device remaining stationary for an extended period. In this work, a stay is equivalent to an activity (e.g., working for a few hours, staying at home overnight, having lunch in a restaurant, exercising in a gym). |
| --- | --- |
| **Activity** | It is a stay labelled for travel purposes. Moving beyond the majority of the research focus on commuting patterns, we endeavoured to identify variable types of daily activities for a wider range of urban applications. Apart from primary activities, i.e., at home and work, we also labelled secondary activities, including education, eating and drinking, shopping (for regular daily grocery shopping, etc. and other shopping like outlets, etc.), entertainment, and others. |
| **Trip** | It is generated by connecting a series of consecutive non-stationary points. A trip means a move from one activity location to another associated with one or multiple travel purposes. |
| **Trip-activity chain** | A series of short trips linked together between activity locations, such as a trip that leaves home, stops to drop off a kid at school, and continues to work. In the context of this work, a trip-activity chain is considered equivalent to a trajectory. |
| **Origin-destination matrix (O-D matrix)** | A matrix summarising counts of trips between defined spatial units (e.g., census tracts). |

**Figure S2 .** Sensitivity analysis of parameters used for clustering


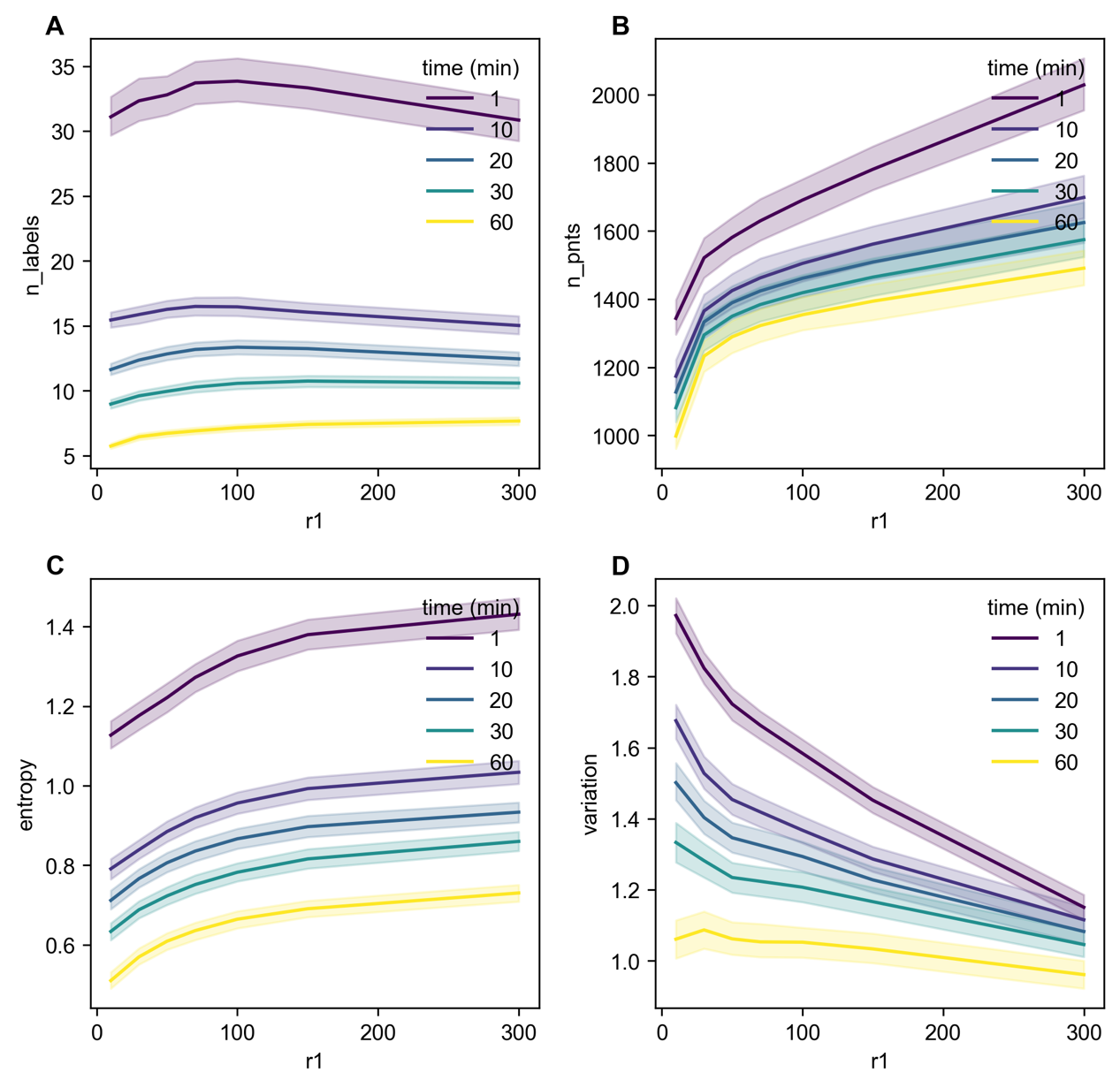


To determine an appropriate spatial threshold $r_{1}$ for identifying stay points, we conducted a sensitivity analysis across four metrics: the number of unique labels (n_labels), total number of stay points (n_pnts), entropy, and variation. As shown in panel A, n_labels initially increases with $r_{1}$, reaching a peak around 80 meters, before declining at larger values. This pattern reflects a trade-off: when $r_{1}$ is too small (e.g., <30 m), minor GPS drift can artificially fragment a single stay into multiple short distance trips, reducing the number of detected stop locations. As $r_{1}$ increases, these artificial fragments are merged, improving location stability and boosting unique label counts. However, beyond a certain point (≈80 m), distinct spatial locations begin to merge, reducing diversity in identified stay points.

Meanwhile, panel B shows that n_pnts (total stays) sharply increases around 25 m and then plateaus, indicating that very small $r_{1}$values result in over-fragmentation, while moderate values consolidate signal noise into coherent stops. Panel C and D confirm this stabilization trend: entropy increases and variation decreases consistently with growing $r_{1}$, supporting that spatial smoothing improves interpretability.

Based on this multi-metric sensitivity analysis, we selected $r_{1}=50$meters as a balanced threshold. It minimizes noise-induced fragmentation, preserves spatial diversity close to its peak, and avoids premature merging of semantically distinct locations. This choice aligns with both signal characteristics and urban-scale interpretability.

**Table S2.** Categories of POIs used in activity labelling

| Activity Types | Activity Location Type |
| --- | --- |
| Education | Primary, secondary, and infant schools, independent and preparatory schools, higher education establishments, other schools such as diving schools, drama schools, language schools, ballet and dance schools, beauty and hairdressing schools, etc. |
| Eating and Drinking | Restaurants, cafes, snack bars, tea rooms, pubs, bars, fish and chip shops, fast food delivery services, etc. |
| Shopping_type1 | Grocers, markets, supermarket chains, Cash and carry, fishmongers, bakeries, etc. |
| Shopping_type2 | Clothing, footwear, jewellery and fashion accessories, books and maps, florists, furniture, lighting, Electrical goods and components, second hand vehicles, etc. |
| Entertainment | Theatre, cinema, recreational, gambling, sport and entertainment services such as gym, etc. |
| Others | The rest of the POIs such as sport and entertainment, health, transport, etc. |

**Supplementary Equation 1. POI-Based Activity Labelling Using the Huff Model**

To assign probable activity types to detected stay points, we applied a probabilistic approach based on the Huff model, which integrates both spatial and temporal factors. The Huff model estimates the probability that an individual stay point is associated with a particular category of Point of Interest (POI) as follows:

$$P_{i,c}= \frac{S_{c}\left( t \right) Max\left( D_{ij}^{-\beta} \right)_{j\in POI\left( c \right)}}{\Sigma_{c^{'} \in C}S_{c^{'}}\left( t \right) Max\left( D_{ik}^{-\beta} \right)_{k\in POI\left( c^{'} \right)}}$$

 Where:

- $P_{i,c}$is the probability that stay point i is associated a specific category c.
- $D_{ij}$is the distance between stay point i and POI j; we use a 500-meter buffer to filter potential POIs.
- $S_{c}\left( t \right)$is a time-dependent weight for POI in category c at time t, reflecting the relevance or popularity of the POI category (Appendix E-2).
- β are empirically defined parameters controlling the influence of attractiveness and distance, respectively.

In our implementation:

- Distance threshold: A 500-meter buffer is applied around each stay point to filter plausible POI candidates.
- Distance term $D_{ij}$: Calculated as the Euclidean distance to the nearest POI within the buffer.
- Attractiveness term $S_{c}\left( t \right)$: Incorporates time-dependent weights, derived from travel surveys and time-use studies, to reflect temporal variations in POI relevance. For instance, workplace POIs are more likely to be associated with morning stays, while retail or leisure POIs are weighted higher during afternoon and evening hours.

The final activity label for each stay point is determined by selecting the POI category with the highest joint probability, integrating both spatial proximity and temporal likelihood.

This approach allows for flexible, interpretable activity labelling while accounting for the known spatiotemporal patterns of human urban behaviour. We acknowledge the following limitations for this model. First, classification errors or missing entries in the POI data propagate directly to activity labels, and mixed-use buildings are inherently difficult to disambiguate, introducing systematic uncertainty; second, our use of Euclidean distance with a fixed 500-m buffer ignores real-world barriers and street-network structure, and results are sensitive to the chosen search radius and distance-decay parameter, with different settings yielding materially different outcomes; third, the time-of-day priors are drawn from external travel and time-use surveys whose representativeness may be limited—they may not reflect all sub-populations or atypical days and can drift over time—so periodic calibration and sensitivity analyses are required.

**Table S3. Time dependent weight** $S_{c}\left( t \right)$ for POI category c in time t

| Start time | Education | Eating and  Drinking | Shopping1 | Shopping2 | Entertainment | Others |
| --- | --- | --- | --- | --- | --- | --- |
| 06:00 – 7:00 | 0.1 | 0.1 | 0.1 | 0.1 | 0.1 | 0.3 |
| 07:00 – 8:00 | 0.7 | 0.7 | 0.5 | 0.1 | 0.1 | 0.3 |
| 08:00 – 9:00 | 0.9 | 0.7 | 0.5 | 0.1 | 0.1 | 0.7 |
| 09:00 – 10:00 | 0.5 | 0.5 | 0.5 | 0.5 | 0.3 | 0.5 |
| 10:00 – 11:00 | 0.3 | 0.5 | 0.5 | 0.7 | 0.5 | 0.7 |
| 11:00 – 12:00 | 0.3 | 0.9 | 0.5 | 0.7 | 0.5 | 0.7 |
| 12:00 – 13:00 | 0.3 | 0.9 | 0.7 | 0.3 | 0.7 | 0.3 |
| 13:00 – 14:00 | 0.3 | 0.7 | 0.7 | 0.5 | 0.5 | 0.3 |
| 14:00 – 15:00 | 0.3 | 0.5 | 0.5 | 0.7 | 0.5 | 0.5 |
| 15:00 – 16:00 | 0.5 | 0.3 | 0.5 | 0.7 | 0.5 | 0.5 |
| 16:00 – 17:00 | 0.3 | 0.5 | 0.5 | 0.7 | 0.5 | 0.5 |
| 17:00 – 18:00 | 0.3 | 0.7 | 0.5 | 0.7 | 0.5 | 0.5 |
| 18:00 – 19:00 | 0.3 | 0.9 | 0.3 | 0.1 | 0.3 | 0.3 |
| 19:00 – 20:00 | 0.3 | 0.9 | 0.7 | 0.1 | 0.7 | 0.3 |
| 20:00 – 21:00 | 0.1 | 0.5 | 0.7 | 0.1 | 0.9 | 0.5 |
| 21:00 – 22:00 | 0.1 | 0.5 | 0.5 | 0.1 | 0.7 | 0.5 |
| 22:00 – 23:00 | 0.1 | 0.3 | 0.5 | 0.1 | 0.5 | 0.5 |
| 23:00 – 24:00 | 0.1 | 0.3 | 0.5 | 0.1 | 0.3 | 0.5 |

**Figure S3.** Hourly user counts of stay location type for each day of the week.


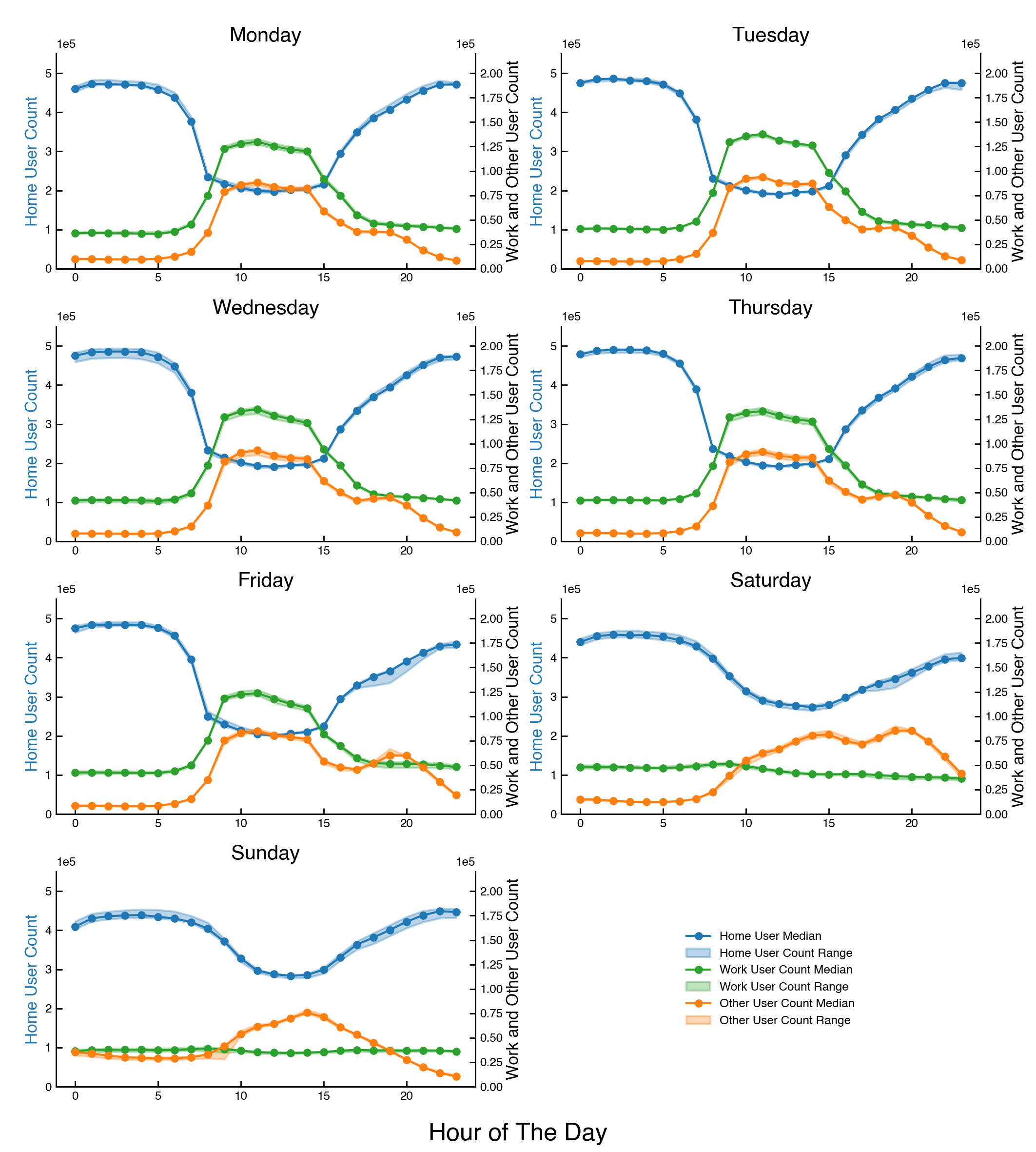


A user is considered to be staying at a location in a given hour only if this user has a stationary activity that covers a continuous period of at least 30 minutes in that hour. Therefore, a user can only be counted to a single type of location in each hour. From the plots, it is evident that home user counts exhibit a distinct diurnal pattern, peaking during the early morning and late evening hours while dipping during typical work hours. Conversely, work user counts show an inverse relationship, with higher counts during standard working hours between 9 AM and 3 PM on weekdays. We also observed significant night and weekend workers. The 'other' user counts demonstrate a more varied pattern, with noticeable peaks during midday on weekdays and early afternoon hours, Friday evenings, weekend afternoons, and Saturday evenings. Overnight stays at other locations are significant on Saturday nights.

**Figure S4.** Data representativeness of the UK population


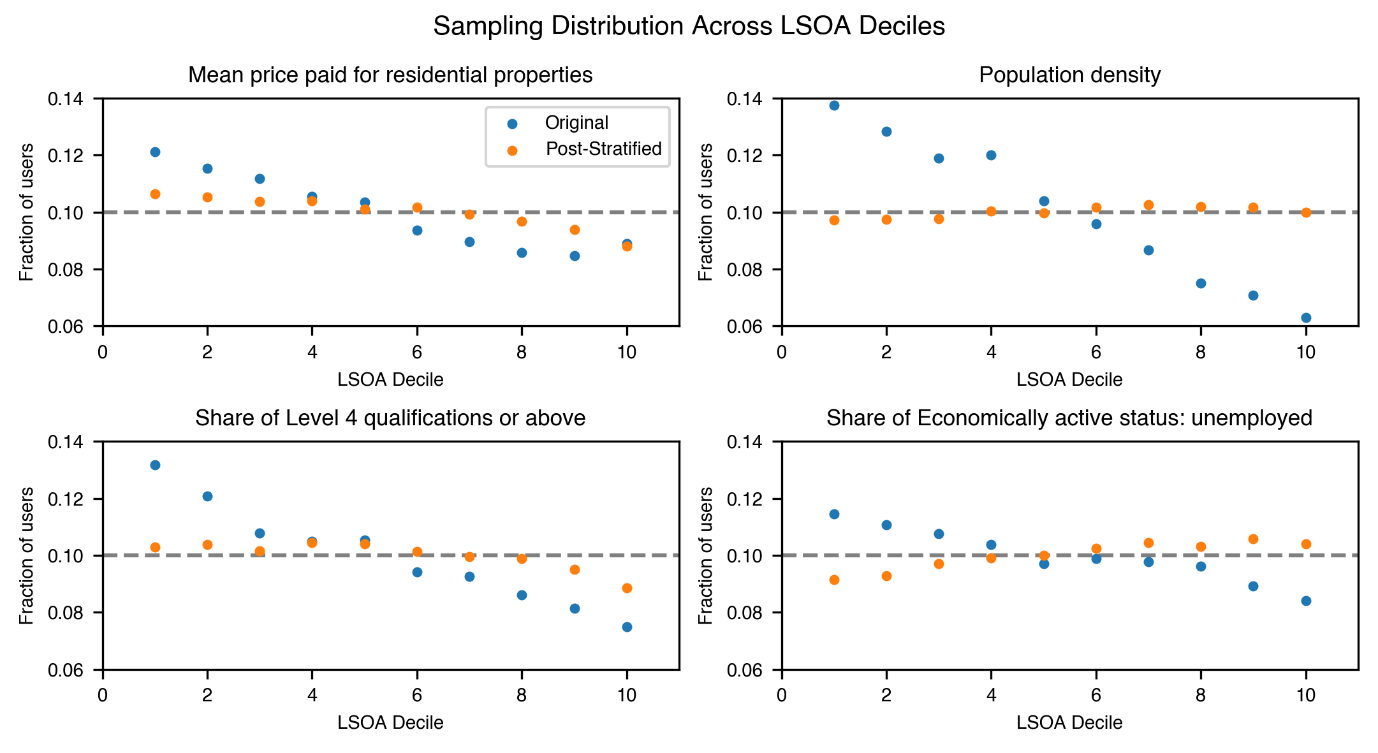


To assess dataset representativeness, we compared the distributions of detected users' home locations from the full datasets with official UK Census indicators at the LSOA level. Figure X presents the proportion of users falling within each decile of four key characteristics: mean property price, population density, share of highly educated residents, and unemployment rate.

If the sample were perfectly balanced, 10% of users would be distributed across each decile. However, the original sample exhibits overrepresentation of users from areas with lower housing prices, lower population density, lower education levels, and lower unemployment rates—reflecting known socioeconomic biases in mobile app data. Following a post-stratification adjustment, the shared sample achieves a more uniform distribution across deciles, improving alignment with the underlying population structure.

**Figure S5.** Agreement between census-based and mobile-phone–based flows.

**
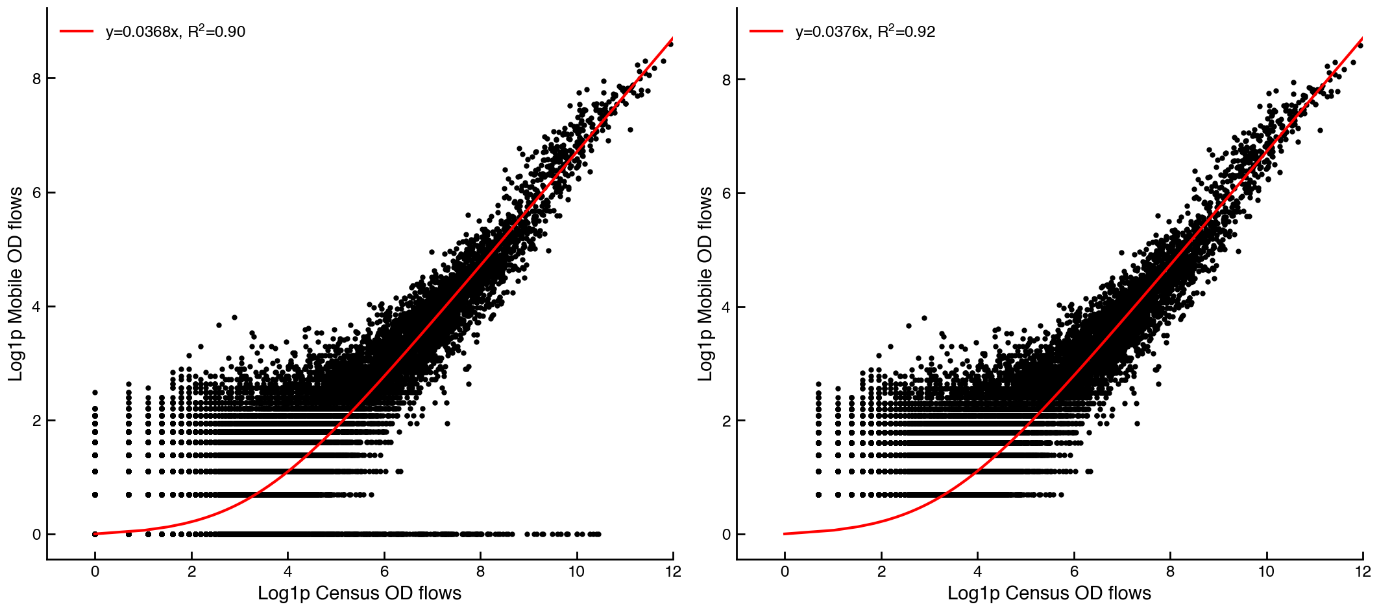
**

Scatter plots of flows with mobile-based on the y-axis and census-based on the x-axis. **Left:** all OD pairs. **Right:** OD pairs with positive flows in both sources (zeros removed). The red line is the OLS fit (equation and (R2) displayed in the panels). Points lie close to the trend and the fit tightens after removing zeros ((R^{2}\approx 0.92)), indicating that the R² gaps in Fig. 4 mainly reflect zero-inflation rather than outliers among large flows.

OD zero breakdown:

(census>0, mobile=0)=30,978;

(census =0, mobile =0)=26,171;

(census =0, mobile >0)=6,369;

(census >0, mobile >0)=31,963.

**Figure S6.** Trip–Population Scaling (β) by MSOA in London


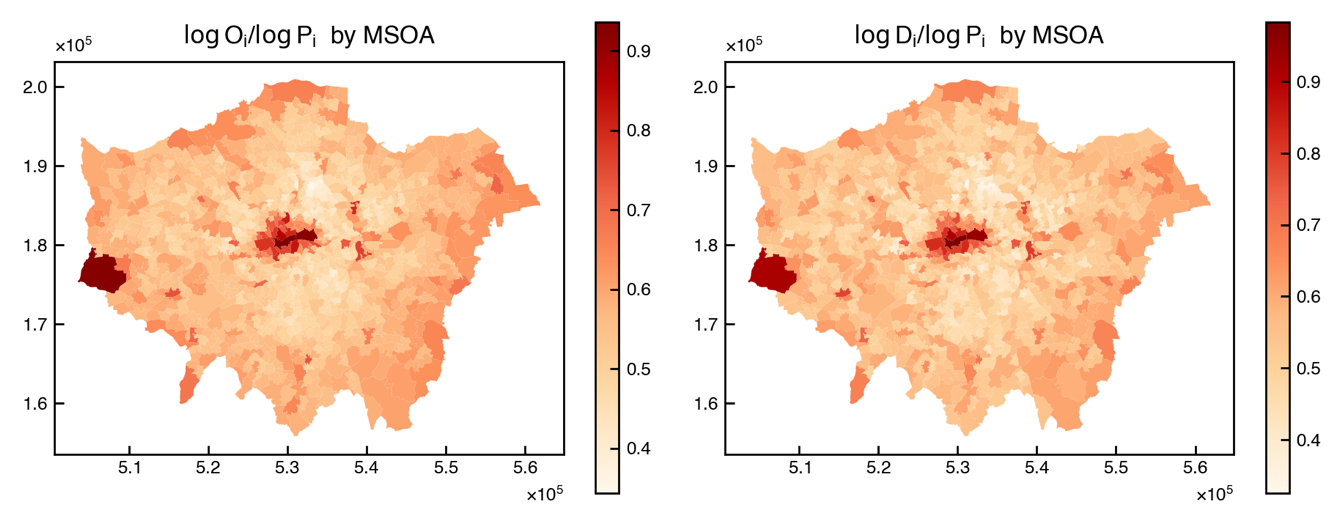


To examine how mobility intensity relates to population, we aggregate the mobile OD to the MSOA level and compute, for each area (i), the total observed outflows ($O_{i}$), inflows ($D_{i}$), and resident population () from census data. We then estimate the scaling exponent (\beta) by fitting the relationships:

($\beta_{i}^{O}=\log O_{i}/\log P_{i}$ and ($\beta_{i}^{D}=\log D_{i}/\log P_{i}$) . Across London all ($\beta$) are below 1, implying sub-linear scaling: trip counts rise more slowly than population. Numerically, ($\beta\approx0.9$) in the central employment core and around Heathrow means that doubling population would raise trips by ($2^{0.9}\approx1.87\times$) (almost proportional), while the widespread ($\beta\approx0.5$) in outer MSOAs implies only ($2^{0.5}\approx1.41\times$).

This pattern is consistent with urban structure and data generation. Central London and Heathrow are major job/transport hubs with intense activity and high device presence, so trips scale almost proportionally with population. In contrast, suburban MSOAs are more residential and dispersed; a larger share of trips crosses MSOA boundaries or occurs with lower activity/device rates, so per-capita trip generation is weaker and remains near 0.5.
